# Potent Inhibition of Human Cytochrome P450 3A4 by Biflavone Components from Ginkgo Biloba and Selaginella Tamariscina

**DOI:** 10.3389/fphar.2022.856784

**Published:** 2022-02-28

**Authors:** Bo Wang, Chao Shi, Lei Feng, Wei Pan, Xiang-Ge Tian, Cheng-Peng Sun, Chao Wang, Jing Ning, Xia Lv, Yan Wang, Qian-Hui Yuan, Rui-Xuan Guan, Hou-Li Zhang, Xiao-Chi Ma, Tong-Hui Ma

**Affiliations:** ^1^ School of Medicine & Holistic Integrative Medicine, Nanjing University of Chinese Medicine, Nanjing, China; ^2^ College of Pharmacy, College of Integrative Medicine, Dalian Medical University, Dalian, China; ^3^ Second Affiliated Hospital, Dalian Medical University, Dalian, China; ^4^ School of Chemistry and Chemical Engineering, Henan Normal University, Xinxiang, China

**Keywords:** cytochrome P450 3A4, herbal-drug interaction, biflavone, metabolism interaction, inhibition mechanism

## Abstract

CYP3A4-mediated Phase I biotransformation is the rate-limiting step of elimination for many commonly used clinically agents. The modulatory effects of herbal medicines on CYP3A4 activity are one of the risk factors affecting the safe use of drug and herbal medicine. In the present study, the inhibitory effects of nearly hundred kinds of herbal medicines against CYP3A4 were evaluated based on a visual high-throughput screening method. Furthermore, biflavone components including bilobetin (7-demethylginkgetin, DGK), ginkgetin (GK), isoginkgetin (IGK), and amentoflavone (AMF) were identified as the main inhibitory components of *Ginkgo biloba* L. (GB) and *Selaginella tamariscina* (P. Beauv.) Spring (ST), which displayed very strong inhibitory effects toward CYP3A4. The inhibitory effects of these biflavones on clinical drugs that mainly undergo CYP3A4-dependent metabolism were evaluated. The *IC*_50_ of GK toward tamoxifen, gefitinib and ticagrelor were found to be of 0.478 ± 0.003, 0.869 ± 0.001, and 1.61 ± 0.039 μM, respectively. These results suggest the potential pharmacokinetic interactions between the identified biflavones and clinical drugs undergoing CYP3A4-mediated biotransformation. The obtained information is important for guiding the rational use of herbal medicine in combination with synthetic pharmaceuticals.

## Introduction

Cytochrome P450 (CYP) enzymes are a large class of heme-thiolate protein superfamily serving the key enzymes in the first-pass metabolism of many foreign substances in humans (Krishna and Shekar, 2005; Omura et al., 2005; Song et al., 2021). Among multiple CYP isoforms, CYP3A4 is mainly expressed in the liver and intestines of adult human, and has a wide substrate spectrum. CYP3A4 participates in the metabolic clearance of more than 50% of commonly used clinical drugs, including tyrosine kinase inhibitors, dihydropyridine calcium antagonists, benzodiazepines sedative-hypnotics, and statins HMG-CoA reductase inhibitors (Niwa et al., 2008; van Waterschoot and Schinkel, 2011). Therefore, CYP3A4 is the key enzyme in the metabolism of many commonly used drugs. It is therefore regarded as one of the most important metabolic enzymes mediating the Phase I biotransformation.

Drugs mainly undergoing CYP3A4 metabolism are more susceptible to adverse drug reactions caused by drug-drug interaction with co-administered pharmaceuticals, and the inhibitory effect of herbal medicines on CYP3A4 can be one of risk factors affecting the safe use of drug and herbal medicine combinations (Fugh-Berman, 2000; Parvez and Rishi, 2019). The components of herbal medicine are complex and highly structurally diverse. Meanwhile, the information on the chemical components and metabolic behavior of herbal medicine is still insufficient (Singh and Zhao, 2017). These factors have brought great challenges to the evaluation and identification of the main inhibitory components of herbal medicine, hindering the pace of research on the inhibition mechanism of herbal medicine.

N-ethyl-1,8-naphthalimide (NEN) is a CYP3A4-specific fluorescent probe developed by our group (Ning et al., 2019). Hydroxylation at C-4 of NEN could trigger “turn-on” fluorescence response. NEN displayed a high specificity toward CYP3A4 over CYP3A5. Furthermore, the C4-hydroxylation of NEN in human liver microsomes can be strongly inhibited by CYP3cide, which is a specific inhibitor of CYP3A4. With ideal selectivity and sensitivity, NEN has been applied to the image of CYP3A4 in complex biological systems (Ning et al., 2019).

In the present study, we employed rapid the screening of the inhibitory action of herbal medicines against CYP3A4 based on a visual high-throughput screening method using NEN. Our results indicate that *Ginkgo biloba* L. (GB) and *Selaginella tamariscina* (P. Beauv.) Spring (ST) display the strongest inhibitory effects toward CYP3A4, and four biflavones were identified as the main inhibitory components of these herbal medicines. Subsequently, the inhibitory effects of these biflavones on clinical drugs mainly undergoing CYP3A4-dependent metabolism were evaluated. Results of these studies suggest the potential interaction between these biflavones and clinical drugs.

## Materials and Methods

### Reagents and Enzymes

The prepared slices of Chinese crude medicines were purchased from Beijing Tongren Tang Co., Ltd. (Dalian, China). Ginkgetin, isoginkgetin, bilobetin, and amentoflavone were purchased from Herbest Biotechnology Co., Ltd. (Baoji, China). N-ethyl-1,8-naphthalimide (NEN) was synthesized by our lab, and was fully characterized by NMR and MS techniques. Midazolam, NADPNa_2_, glucose-6-phosphate dehydrogenase, and D-glucose-6-phosphate (G-6-P) were obtained from Sigma-Aldrich (St. Louis, MO, United States). The pooled human liver microsomes (HLM) were purchased from Bioreclamation IVT (MD, United States).

### Preparation of Herbal Extracts

Medical materials were ground to coarse powder, and then immersed into 10 volumes of 95% ethanol. Subsequently, the powdered materials were extracted twice with ultrasound (600 W) for 30 min. The filtered extracts were enriched by a rotary vacuum evaporator. The extracts were dissolved in DMSO as the stock solution (20 mg/ml) for the subsequent.

### Inhibition Assays of NEN 4-Hydroxylation

The primary incubation mixture contains HLM, potassium phosphate buffer (pH 7.4, 100 mM), G-6-P (10 mM), G-6-P dehydrogenase (1 U/ml), MgCl_2_ (4 mM), NEN (50 µM), extracts of herbal medicine (20 µg/ml), or components. The incubation duration and HLM protein concentrations were 30 min and 0.05 mg/ml, respectively. The reaction was initiated by the addition of NADP^+^ and terminated by the addition of 100 µL ice-cold acetonitrile (ACN). NEN 4-hydroxylation were utilized as the probe reaction for CYP3A4, and fluorescence image of 96-well microplates were measured by a fluorescent image analyzer (GE Amersham Typhoon, Sweden) under the excitation wavelength of 488 nm (*E*_m_: 570 ± 15 nm). The formation of NEHN was evaluated by the quantitative analysis of fluorescence signal for individual wells of microplates using the equipment supporting software. The percentage residual activity was calculated from percentage relative value of NEHN formation rate.

### Identification of the Inhibitory Components of Herbal Medicines

The chemical fingerprints of *Ginkgo biloba* L. (GB) and *Selaginella tamariscina* (P. Beauv.) Spring (ST) were analyzed based on UPLC with a photodiode array detector.

Fractions were collected with an Acquity UPLC system (Waters, Milford, MA, United States) equipped with a photodiode array detector (2998), an auto sampler, a quaternary gradient pump, and a column compartment. An AQCUITY UPLC HSS C18 (100 mm × 2.1 mm, 1.8 μm, Waters) analytical column was used and kept at 30°C. The mobile phase consisted of 0.3% trifluoroacetic acid in water (A) and Methanol (B). The following gradient conditions was used: 0.0–5.0 min, 85% A, 5.0–38.0 min, 85–20% A, 38.0–41.00 min, 20% A, 41.0–44.0 min, 20–85% A, 44.0–47.0 min, 85% A for analysis of GB extract; 0.0–3.0 min, 65% A, 3.0–10.0 min, 65%–10% A, 10.0–15.00 min, 10% A, 15.0–18.0 min, 10%–65% A, 18.0–20.0 min, 65% A for analysis of ST extract. The precision of the fingerprinting method was previously confirmed by comparison of the HPLC spectrum mainly considering the relative retention time and peak area. Then, the LC fractions of crude extract (20 mg/ml, 10 μL) were collected consecutively at 30 or 60 s intervals, where the flow rate was set at 0.3 ml/min.

All LC fractions were dried *in vacuum*, and redissolved in DMSO. The fractions were evaluated for their activity moderating effect for CYP3A4 based on the high-throughput screening method. Then, the target fractions underwent further preparation and purification by semi-preparative HPLC.

### Inhibition Kinetic Characterization

Varying concentrations of DGK, GK, IGK, and AMF incubated for 30 min with varying concentrations of NEN (15–150 µM). The high-throughput screening assay was performed, and the inhibition curve was fitted with GraphPad Prism (Version 8.0). The inhibition type was confirmed based on the R square of the following four equations and the Dixon and Lineweaver–Burk plots. The inhibition constant (*K*_i_) values were determined using the equations for competitive inhibition (Eq. 1), noncompetitive inhibition (Eq. 2), uncompetitive inhibition (Eq. 3), and mixed inhibition (Eq. 4).

$$v=\frac{V_{max}S}{K_{m}\left(1+\frac{I}{K_{i}}\right)+S}$$ (1)

$$v=\frac{V_{max}S}{\left(K_{m}+S\right)\left(1+\frac{I}{K_{i}}\right)}$$ (2)

$$v=\frac{V_{max}S}{S\left(1+\frac{I}{K_{i}}\right)+K_{m}}$$ (3)

$$v=\frac{V_{max}S}{K_{m}\left(1+\frac{I}{K_{i}}\right)+S\left(1+I/\alpha K_{i}\right)}$$ (4)

### Inhibition Assays of Clinical Drugs

Varying concentrations of DGK, GK, IGK, and AMF incubated for 30 min with gefitinib (10 μM), tamoxifen (20 μM), ticagrelor (30 μM), respectively (Wang et al., 2020). The ExionLC AD HPLC system consisted of an autosampler, a quaternary delivery system and a degasser. The chromatograph was equipped with a Luna Omega PS C18 (2.1 × 100 mm, 3 μm) analytical column, and the mobile phase consisted of 0.1% formic acid aqueous solution (A) and ACN (B), and were conducted with a 0.30 ml/min flow rate as follow for the gradient program: 0–1.5 min (10–10% B), 1.5–3.0 min (10–45% B), 3.0–5.0 min (45–90% B). An AB MDS Sciex QTRAP 5500 Mass Spectrometer equipped with an ESI source was used to analyze the target substances. The positive mode was used with N-demethylated tamoxifen (358.0→58.0) and defluorinated gefitinib (445.0→128.0). The relative parameters were set as follows: ion spray voltage, 5500 V; temperature, 550°C; curtain gas (CUR) flow, 35 L/min; gas1 and gas2 (nitrogen), 45 and 55 psi. The negative mode was used with dehydroxyethoxylated ticagrelor (477.2→361.1). The relative parameters were set as follows: ion spray voltage, -4500 V; temperature, 550°C; curtain gas flow, 35 L/min; gas1 and gas2, 50 and 50 psi. For other parameters, please refer to Supplementary Table S1 in the supplementary data.

## Results

### Inhibition Screening of Herbal Medicine Extracts Toward CYP3A4

**FIGURE 1:**
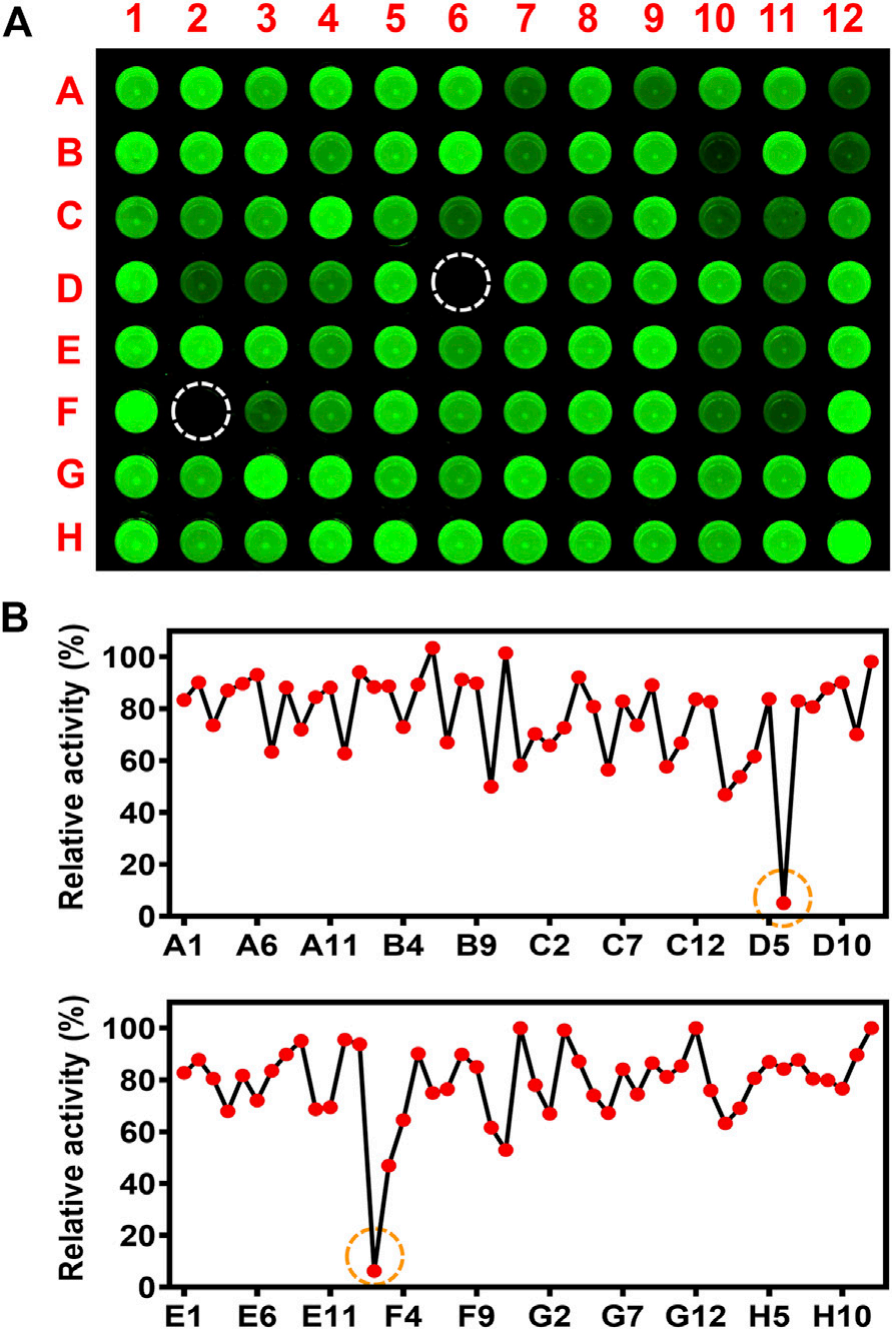
High-throughput inhibition screening of inhibitory effects of herbal medicine towards CYP3A4. **(A)** Inhibition of CYP3A4 by herbal medicine extracts was determined based of their effect on the intensity of fluorescence at 570 ± 15 nm (λ_ex_: 488 nm, λ_em_: 570 ± 15 nm, F12, G12, and H12 are control samples treated with solvent DMSO. **(B)** Quantitative analyses of inhibitory effects of herbal medicine towards CYP3A4. The residual activity was calculated by the volume ratio of herbal medicine and control samples (F12, G12, and H12).

The inhibitory effects of 93 herbal medicines toward CYP3A4 were preliminary evaluated using the high-throughput screening system constructed with CYP3A4 selective fluorescent probe NEN (Figure 1). Inhibition of CYP3A4 by herbal medicine extracts was determined based of their effect on the intensity of fluorescence at 570 ± 15 nm that was excited at 488 nm. Visual observation of the fluorescence images of the multi-well plates reveals different degree of modulatory effect of herbal medicines toward CYP3A4 activity. *Ginkgo biloba* L. (GB, F2) and *Selaginella tamariscina* (P. Beauv.) Spring (ST, D6) exhibited ultra-strong inhibitory effects toward CYP3A4 resulting in virtual elimination of the fluorescence signal in the corresponding wells of the 96-wells microplate. Quantitative analysis of the inhibitory effects of herbal medicine towards CYP3A4 demonstrate that the residual activities of CYP3A4 after treatment with the extracts of GB and ST were 6.3 and 5.0%, respectively (Supplementary Table S2). The quantitative results were consistent with the visually observed trend of the modulatory effects toward CYP3A4 activity. Additionally, three herbal medicine extracts exhibited relatively strong inhibitory effects towards CYP3A4 with residual activity less than or equal to 50%, namely *Tribulus terrestris* L. (D2), *Areca catechu* L. (B10), and *Fissistigma glaucescens* (Hance) Merr. (F3).

### Identification of the Inhibitory Components of Herbal Medicine

**FIGURE 2:**
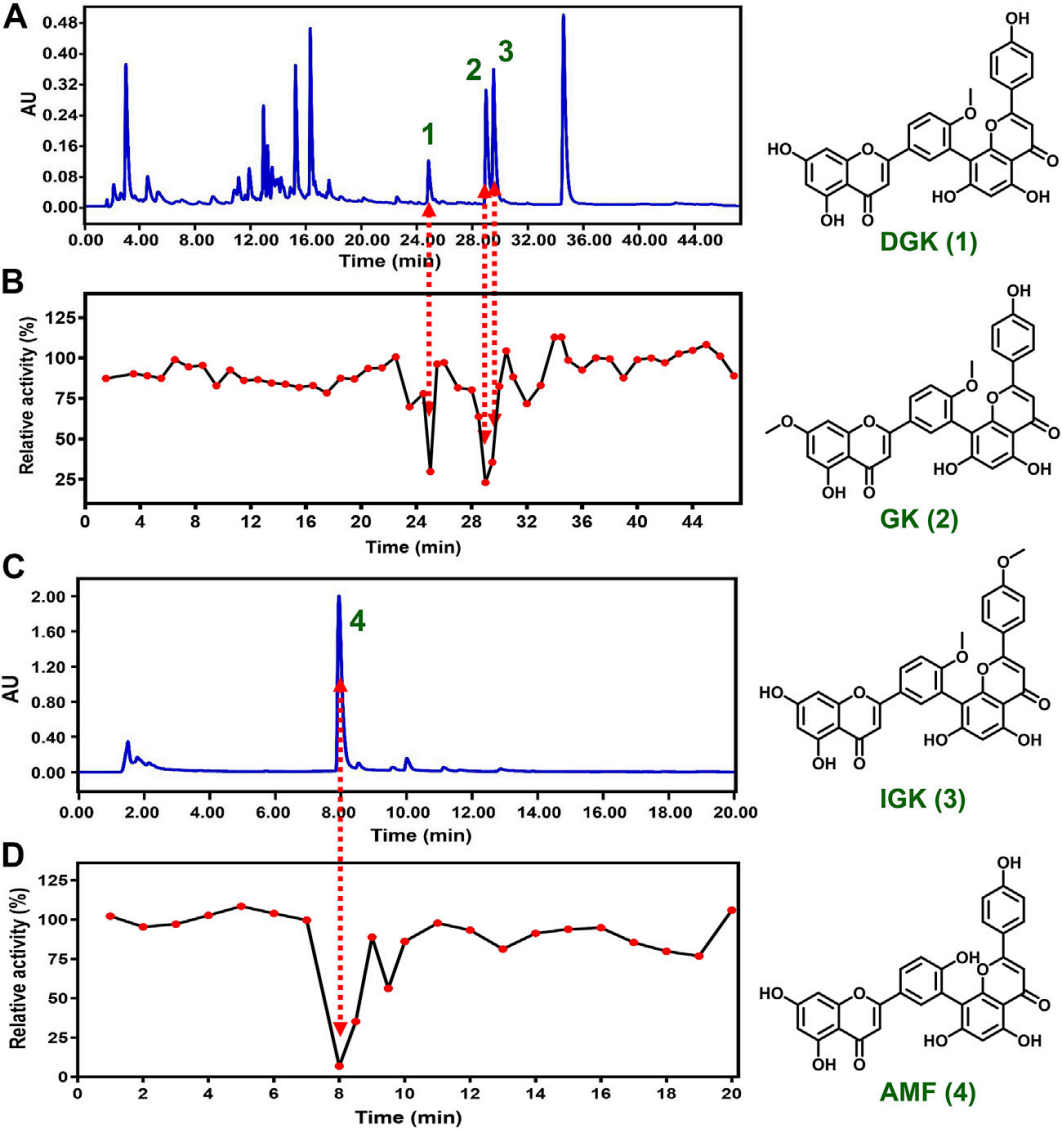
Identification the inhibitory components toward CYP3A4 in herbal medicines. LC-UV fingerprint of GB **(A)** and ST **(C)** extract, and the corresponding CYP3A4 inhibition profile of LC fractions **(B, D)**.

We attempted to reveal the inhibitory components of GB and ST since they exhibited strong inhibitory effect towards CYP3A4. However, the complexity and structural diversity of the two herbal medicines are very high, especially GB. In the present study, a combination of chemical fingerprinting-guided LC fraction collection and screening of the inhibitory potential was used to identify the inhibitory components of herbal medicine. The LC fractions of GB and ST were collected consecutively at appropriate time intervals (30 or 60 s) based on the reliable LC method which was confirmed by reproducible relative retention time of the main components of herbal medicine (RSD < 3%). Subsequently, the inhibitory components of GB and ST toward CYP3A4 were successfully identified by bioassay guided LC fractions studies utilizing high-throughput visualization screening. For GB, three inhibitory components were picked out from a comparison of the UPLC-UV fingerprint with the profile of CYP3A4 inhibition (Figure 2). Then, three key components of GB were prepared by bioassay-guided LC fractionation and identified as bilobetin (7-demethylginkgetin, DGK), ginkgetin (GK), and isoginkgetin (IGK), respectively, according to the comparison of the LC retention times, spectral data, and the mass data of isolated compounds and authentic standards (Wang et al., 2015). Amentoflavone (AMF), meanwhile, was identified as the main component contributing to the strong inhibitory effect of ST (Table 1).

**TABLE 1:** Identification and characterization of inhibitory components with potent CYP3A4 inhibitory effects in GB and ST.

Peak no.	t_R_ (min)	Pseudo molecular ion [M + H]^+^	Product ions (ESI^+^)	MW	Formula	Identification
1	24.8	553.1119	553 [M + H]^+^	552	C_31_H_20_O_10_	DGK
			521 [M + H-CH_3_OH]^+^			
			403 [M + H-C_8_H_6_O-CH_3_OH]^+^			
			297 [M + H-C_8_H_6_O-C_2_H_2_O-C_3_O_2_-CO]^+^			
			153 [C_7_H_4_O_4_]^+^			
			121 [C_7_H_6_O_2_]^+^			
2	28.8	567.1286	567 [M + H]^+^	566	C_32_H_22_O_10_	GK
			535 [M + H-CH_3_OH]^+^			
			417 [M + H-C_8_H_6_O-CH_3_OH]^+^			
			167 [C_8_H_6_O_4_]^+^121 [C_7_H_6_O_2_]^+^			
3	29.3	567.1321	567 [M + H]^+^	566	C_32_H_22_O_10_	IGK
			535 [M + H-CH_3_OH]^+^			
			153 [C_7_H_4_O_4_]^+^			
			135 [C_7_H_2_O_3_]^+^			
4	7.9	539.0984	539 [M + H]^+^	538	C_30_H_18_O_10_	AMF
			403 [M + H-C_8_H_6_O-H_2_O]^+^			
			377 [M + H-C_9_H_6_O_3_]^+^			
			153 [C_7_H_4_O_4_]^+^			
			121 [C_7_H_6_O_2_]^+^			

### The Concentration-Dependent Inhibitory Effects of Biflavones Against CYP3A4

**FIGURE 3:**
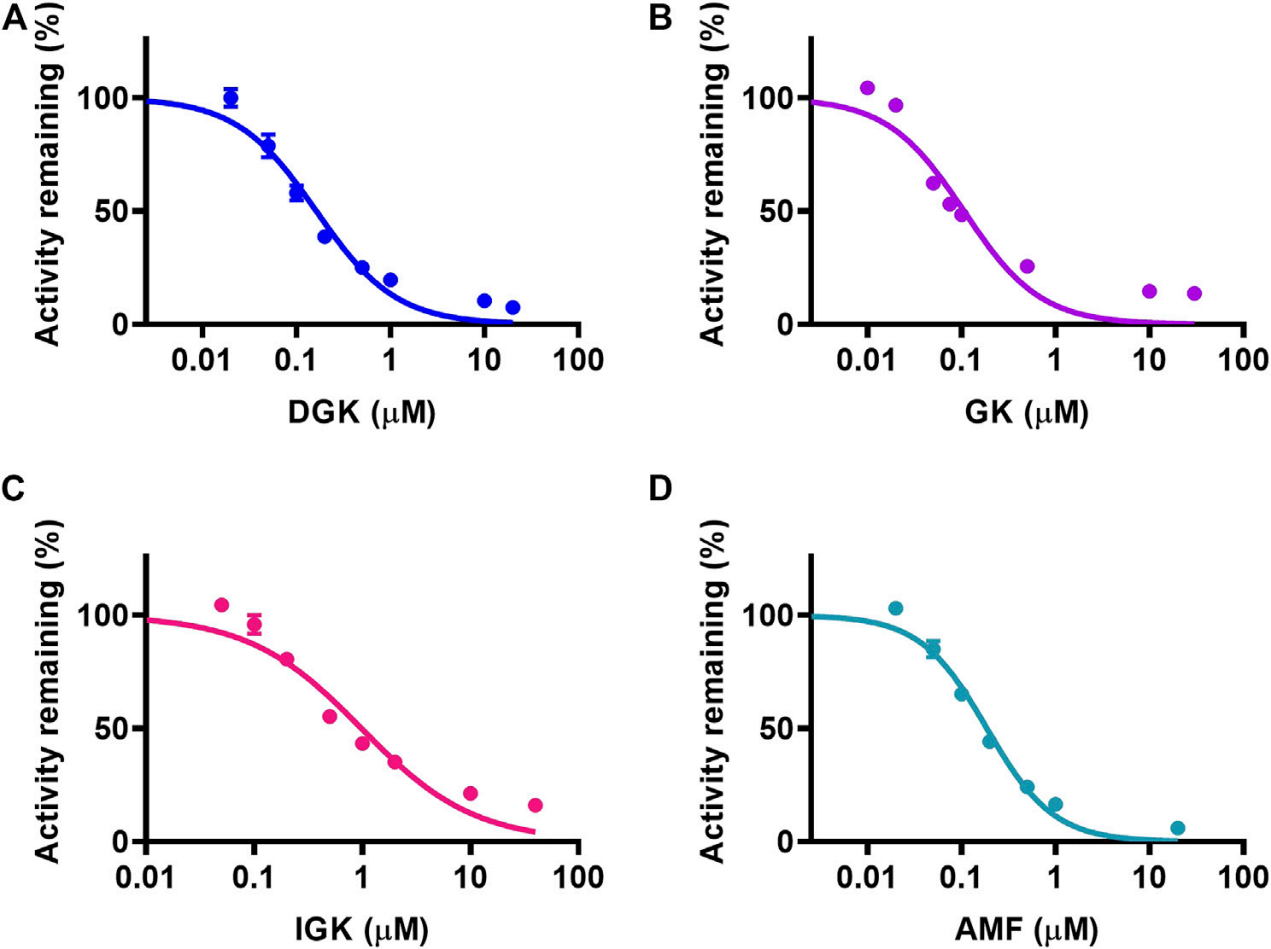
The concentration-dependent inhibitory effects of four identified biflavones against NEN-4-hydroxylation in HLM. **(A)** DGK, **(B)** GK, **(C)** IGK, and **(D)** AMF. Data are shown as the mean ± S.D. (*n* = 3). Each line represents the line of best fit to the data.

The concentration range of DGK, GK, IGK, and AMF was used to depict the inhibitory plot and characterize the inhibitory effects of four identified biflavones toward CYP3A4. As shown in Figure 3, all of the four biflavones can potently inhibit the formation of N-ethyl-4-hydroxy-1,8-naphthalimide (NEHN) in HLM in a dose-dependent manner. The *IC*_50_ values of DGK, GK, IGK, and AMF against CYP3A4 were evaluated as 0.162 ± 0.022, 0.106 ± 0.004, 0.982 ± 0.006, and 0.186 ± 0.004 μM, respectively. These findings suggested that the four biflavones exhibited a significant inhibition activity towards CYP3A4 and further confirmed the above conclusion that DGK, GK, IGK, and AMF were the main inhibitory components of GB and ST, respectively.

**FIGURE 4:**
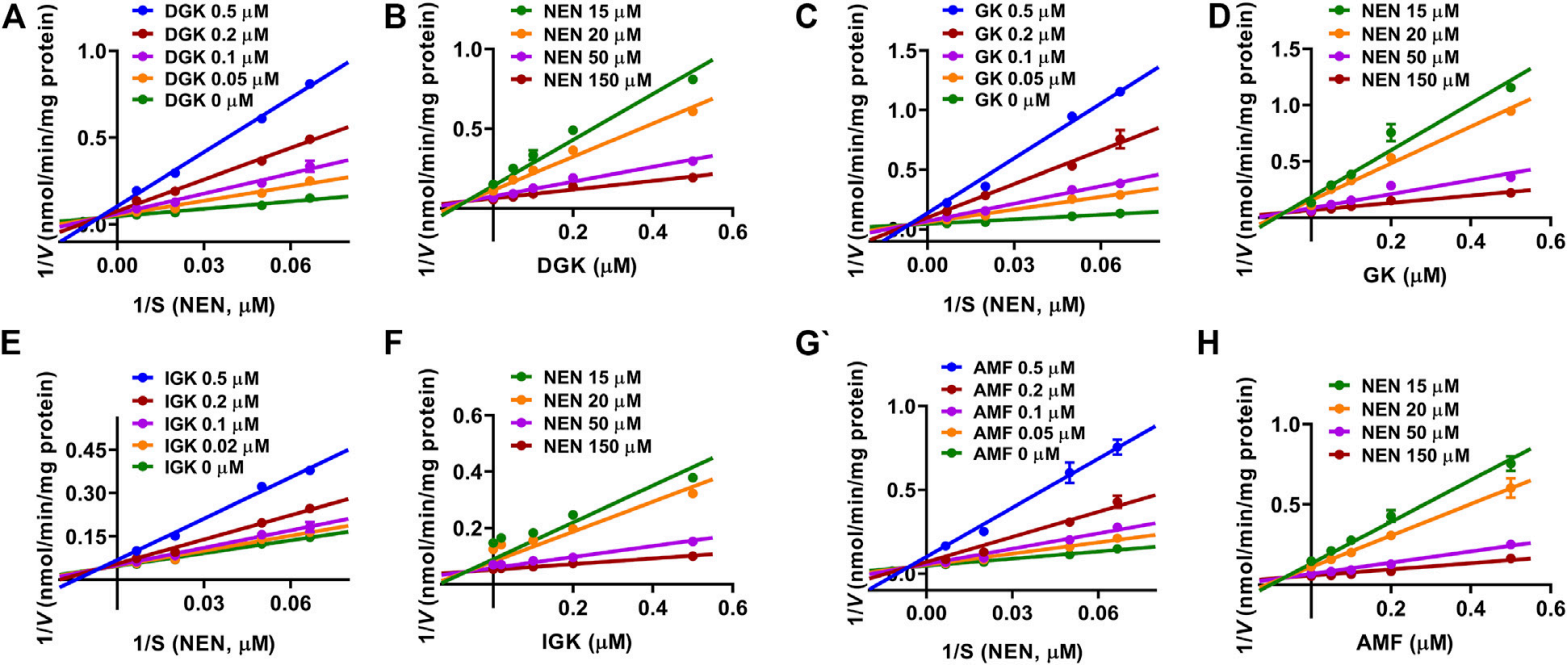
The Lineweaver–Burk (Left) and Dixon (Right) plots of four identified biflavones. **(A, B)** DGK, **(C, D)** GK, **(E, F)** IGK, **(G, H)** AMF. Data are shown as the mean ± S.D. (*n* = 3). Each line represents the line of best fit to the data.

Given that all of the four identified biflavones displayed strong inhibitory effects toward CYP3A4, the inhibition constant (*K*_i_) values of DGK, GK, IGK, and AMF toward CYP3A4 were further determined. As shown in Figure 4A, DGK displayed mixed reversible inhibition against NEN 4-hydroxylation, and the *K*_i_ value was determined to be of 0.070 ± 0.018 μM. Correspondingly, the inhibition profiles of GK, IGK, and AMF were all fitted to mixed reversible inhibition equation with the *K*_i_ values of 0.034 ± 0.007, 0.278 ± 0.087, and 0.101 ± 0.030 μM (Table 2). These results demonstrated that DGK, GK, IGK, and AMF functioned as strong inhibitors of CYP3A4 with the *K*_i_ values ranging from 0.034 to 0.278 µM (Table 2).

**TABLE 2:** Inhibition parameters of inhibitory constitutes toward CYP3A4 in human liver microsomes.

Biflavone	*K*_i_ (µM)	Type of inhibition	ɑ^a^	Goodness of fit (R^2^)
DGK	0.070 ± 0.018	Mixed-inhibition	2.98	0.9825
GK	0.034 ± 0.007	Mixed-inhibition	4.56	0.9859
IGK	0.278 ± 0.087	Mixed-inhibition	2.79	0.9808
AMF	0.101 ± 0.030	Mixed-inhibition	5.55	0.9758

a The α value represent the degree to which the binding of the inhibitor influence the binding between enzyme and substrate. When α is greater than 1, the mixed-inhibition model is close to competitive inhibition.

### The Inhibitory Effects of Biflavones Against the Metabolism of Clinical Drugs

**FIGURE 5:**
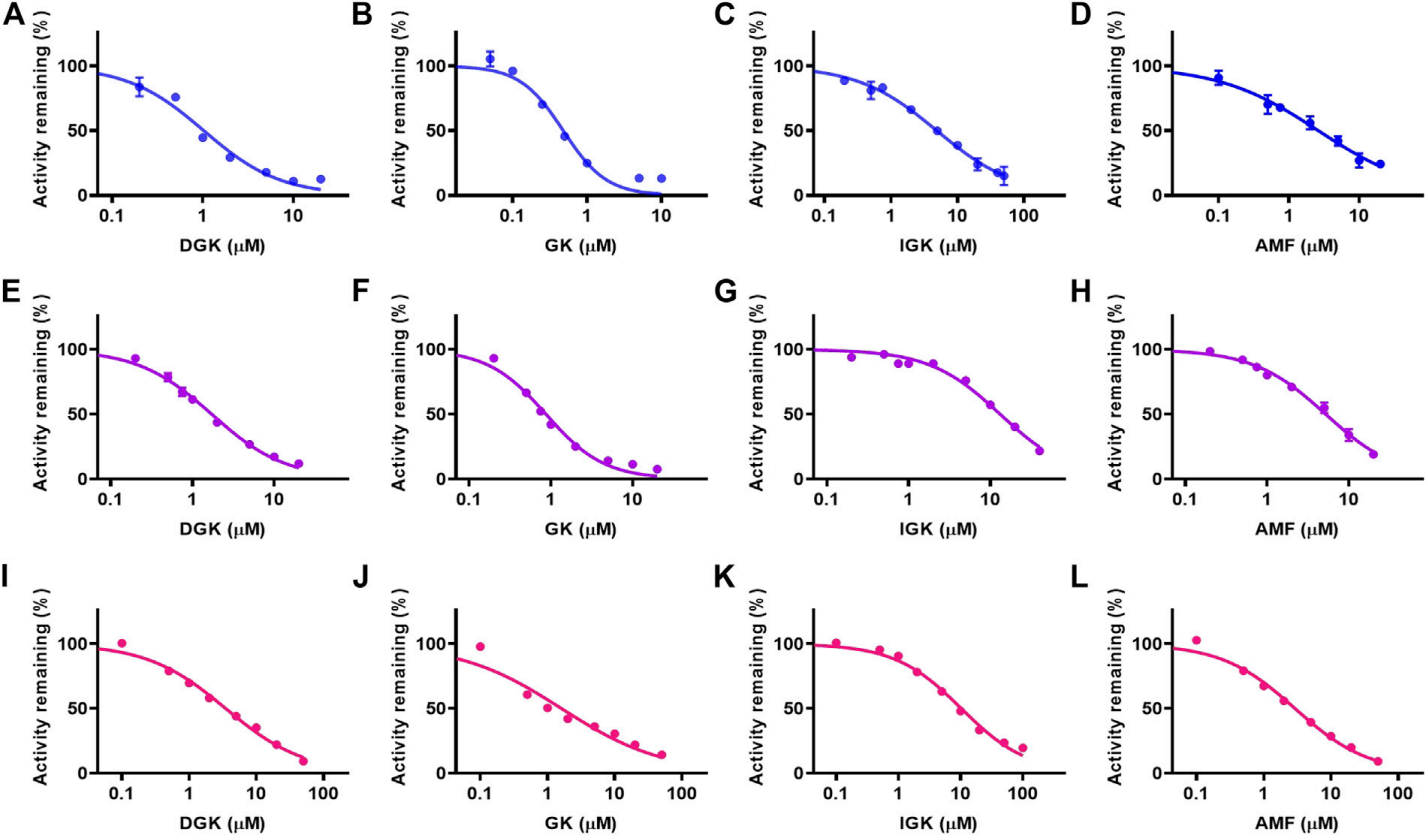
The concentration-dependent inhibitory effects of four identified biflavones against CYP3A4-mediated oxidation of tamoxifen **(A–D)**, gefitinib **(E–H)** and ticagrelor **(I–L)** in HLM. **(A, E, I)** DGK, **(B, F, J)** GK, **(C, G, K)** IGK, **(D, H, L)** AMF. Data are shown as the mean ± S.D. (*n* = 3). Each line represents the line of best fit to the data.

The CYP3A4-mediated potential interactions between DGK, GK, IGK, AMF, and clinical drugs were investigated. The selective estrogen receptor modulator tamoxifen, tyrosine kinase inhibitor gefitinib, and P2Y12 platelet inhibitor ticagrelor, which mainly undergo CYP3A4 metabolism (Desta et al., 2004; Li et al., 2007; Zhou et al., 2011), were subjected to our study of their potential drug interaction profiles. As shown in Figure 5, the four biflavones potently inhibit the CYP3A4-mediated oxidation of three clinical drugs in HLM in a dose-dependent manner. The half-inhibition concentration values for the four biflavones are shown in Table 3. The *IC*_50_ values for GK against tamoxifen and gefitinib was 0.478 ± 0.003 and 0.869 ± 0.001 µM, respectively. It was found that GK exhibited the strongest inhibitory effect towards all three clinical drugs. It is followed by DGK, AMF, and IGK; this order is consistent with that determined in the studies with fluorescent probe NEN.

**TABLE 3:** *IC*_50_ of biflavones toward clinical drugs.

	*IC*_50_ ± SD (µM)			
	DGK	GK	IGK	AMF
Tamoxifen	1.02 ± 0.088	0.478 ± 0.003	4.66 ± 0.236	2.72 ± 0.093
Gefitinib	1.72 ± 0.001	0.869 ± 0.001	13.1 ± 0.415	5.20 ± 0.527
Ticagrelor	3.50 ± 0.034	1.61 ± 0.039	10.2 ± 0.382	2.92 ± 0.047

## Discussion

Nowadays, herbal-drug interactions has become increasingly important due to the wide application of herbal medicines and their important role in the treatment and management of various diseases. Therefore, the effective evaluation of the interactions between herbal medicines and chemical drugs has become necessary to ensure their safe use. According to statistics, about 15% of patients take herbal medicines while receiving chemical drug treatment, and 40% of them exhibit adverse drug interactions, and the incidence rate is as high as 54% in the elderly group (Fugh-Berman, 2000; Bush et al., 2007). The cause of these instances is the high incidence of drug interactions at the metabolic level (Gallo et al., 2019; Parvez and Rishi, 2019). Specifically, when the key metabolic process of a drug is severely interfered by other drugs or components of herbal medicine, the concentration of drug may increase and break the “safety window”, and result in an adverse reaction of drugs. In the present study, the CYP3A4-mediated herbal-drug interaction was investigated considering the important role of CYP3A4 in the metabolism of a variety of clinical drugs.

Among the screened herbal medicines, GB and ST showed strong inhibitory effects on CYP3A4. *Tribulus terrestris* L. (D2), *Areca catechu* L. (B10), *Fissistigma glaucescens* (Hance) Merr. (F3) and *Isatis indigotica* Fort. (F11) displayed a moderate inhibitory effect toward CYP3A4, while the inhibitory effect of the remaining herbal medicines was insignificant. It is noteworthy that GB and *Isatis indigotica* Fort. are the most frequently used herbal medicines in Asia. Patients with ischemic stroke and neurological disorders used GB as a remedy (Ji et al., 2020), and *Isatis indigotica* Fort. is one of the few well-recognized traditional herbs with anti-inflammatory, antibacterial and antiviral effects (You et al., 2009; Chen et al., 2021). Additionally, *Tribulus terrestris* L. is currently approved as a dietary supplement due to its pro-sexual and androgen enhancing effects (Ștefănescu et al., 2020). The above information suggests that researchers should pay close attention to the potential CYP3A4-mediated HDI of herbal medicines such as GB, *Isatis indigotica* Fort., *Tribulus terrestris* L. Our results provide important information for guiding the rational combination of herbal medicines and synthetic pharmaceuticals.

Previous research reports on interactions between GB and CYP indicated that flavonol aglycones such as kaempferol, quercetin, and apigenin were the major CYP3A4 inhibitory components in GB (von Moltke et al., 2004). The discrepancy with the present results may be due to the fact that the previous investigation used only 29 isolated standard substances in GB and lacked any systematic evaluation of biflavone components of GB. In the present study, we observed a significant inhibitory effect of 20 μg/mL GB extract on CYP3A4, and demonstrated an important inhibitory effect of DGK, GK, and IGK towards CYP3A4 with the use of high-throughput inhibition screening that employs NEN as a fluorescent probe for CYP3A4 activity. Therefore, these biflavonoids were identified as the major CYP3A4 inhibitory components of GB. The combination of visual screening with LC fraction tracking methods for the identification of inhibitory components used in this study takes into account both the composition of herbal medicines and the inhibitory effect of its individual components. This is an important advantage over the commonly used method of identification of inhibitory components of herbal medicines that uses a limited range of isolated and identified standard substances.

Biflavonoids are a class of compounds with outstanding anti-inflammatory and antioxidant biological activities, and have anti-microbial, anti-tumor and neuroprotective effects (Gontijo et al., 2017; Menezes and Diederich, 2021). In a variety of animal disease models, the excellent efficacy of diflavonoids has been confirmed (Rong et al., 2019; Su et al., 2019). The inhibitory effects of diflavonoids against CYP3A4 should be further investigated, especially with clinical drugs commonly co-administered with these diflavones. Hence, we evaluated the inhibitory effects of diflavonoids toward three clinical drugs that mainly undergo CYP3A4-mediated metabolism including anticoagulant agent ticagrelor, anti-tumor drug tamoxifen, and gefitinib. We found that the three abundant diflavonoids in GB, namely DGK, GK and IGK, strongly inhibited the metabolism of ticagrelor, with *IC*_50_ of 3.50 ± 0.034, 1.61 ± 0.039, and 10.2 ± 0.382 μM, respectively. Notably, the GB extract is commonly used for treatment in patients exhibiting angina pectoris, myocardial infarction and ischemic stroke, and for improving cardiac function, myocardium microenvironment and neurological function (Ji et al., 2020). Therefore, special attention should be paid to the co-administration of GB extract and ticagrelor. Furthermore, the anticancer effects of biflavonoids including AMF has been widely recognized in the past few years (Kim et al., 2020; Aliyev et al., 2021). The potential of AMF to applications as an anti-cancer drug suggests an importance of evaluation of the metabolic interaction between tamoxifen, gefitinib, and AMF. *IC*_50_ values for the effect of AMF on the oxidation of tamoxifen and gefitinib are equal to 2.72 ± 0.093 and 5.20 ± 0.527 μM, respectively. Our results confirmed a significant inhibitory effects of the diflavonoids on the clinical drugs under study, and also pointed out that more attention should be paid to the herbal-drug interactions caused by the CYP3A4-mediated metabolic interaction between clinical drugs with biflavonoids and the respective herbal medicines.

## Data Availability

The original contributions presented in the study are included in the article/Supplementary Material, further inquiries can be directed to the corresponding authors.
