# Supplementary material for: Potent Inhibition of Human Cytochrome P450 3A4 by Biflavone Components from Ginkgo Biloba and Selaginella Tamariscina
Source: Front Pharmacol. 2022 Feb 28;13:856784. doi: 10.3389/fphar.2022.856784 (PMC8920304; doi:10.3389/fphar.2022.856784)
Supplement: Supplementary file 1 [file DataSheet1.docx]

Supplementary Material

## Table S1. LC/MS analysis parameters of analytes.

| Analytes | Transition (m/z) | DP | EP | CE | CXP |
| --- | --- | --- | --- | --- | --- |
| Defluorinated gefitinib | 445.0→128.0 | 90 | 10 | 15 | 12 |
| N-demethylated tamoxifen | 358.0→58.0 | 90 | 10 | 20 | 12 |
| Dehydroxyethoxylated ticagrelor | 477.2→361.1 | -90 | -9 | -28 | -16 |

**Table S2**. Screening data for CYP3A4 inhibition by herbal medicines

|  | NO. | Herbal medicine | Residual activity (%) |  | NO. | Herbal medicine | Residual Activity (%) |
| --- | --- | --- | --- | --- | --- | --- | --- |
| **A1** | 1 | Salviae Miltiorrhizae Radix Et Rhizoma | 83.3 | **E1** | 49 | Picrorhizae Rhizoma | 82.8 |
| **A2** | 2 | Crataegi Fructus | 90.2 | **E2** | 50 | Sinapis Semen | 87.9 |
| **A3** | 3 | Carthami Flos | 73.6 | **E3** | 51 | Pruni Semen | 80.5 |
| **A4** | 4 | Morindae Officinalis Radix | 87.1 | **E4** | 52 | Loquat | 67.9 |
| **A5** | 5 | Fructus Malvae Verticillatae | 89.7 | **E5** | 53 | Menthae Haplocalycis Herba | 81.7 |
| **A6** | 6 | Cynomorii Herba | 93.2 | **E6** | 54 | Eupatorii Herba | 72.0 |
| **A7** | 7 | Angelicae Dahuricae Radix | 63.4 | **E7** | 55 | Ginseng Radix Et Rhizoma Rubra | 83.5 |
| **A8** | 8 | Polygonati Rhizoma | 88.2 | **E8** | 56 | Fraxini Cortex | 89.9 |
| **A9** | 9 | Rhei Radix Et Rhizoma | 72.0 | **E9** | 57 | Rehmanniae Radix Praeparata | 95.1 |
| **A10** | 10 | Chinese Waxgourd Seed | 84.5 | **E10** | 58 | Mume Fructus | 68.7 |
| **A11** | 11 | Erigerontis Herba | 88.2 | **E11** | 59 | Inulae Flos | 69.5 |
| **A12** | 12 | Fritillariae Thunbergii Bulbus | 62.7 | **E12** | 60 | Imperatae Rhizoma | 95.5 |
| **B1** | 13 | Sterculiae Lychnophorae Semen | 94.1 | **F1** | 61 | Hordei fructus Germinatus | 93.8 |
| **B2** | 14 | Polygonati Odorati Rhizoma | 88.3 | **F2** | 62 | Ginkgo Folium (*Ginkgo biloba* L.) | 6.3 |
| **B3** | 15 | Paeoniae Radix Alba | 88.7 | **F3** | 63 | Root of Glaucescent Fissistigma  (*Fissistigma glaucescens* (Hance) Merr.) | 46.9 |
| **B4** | 16 | Nelumbinis Rhizomatis Nodus | 72.9 | **F4** | 64 | Spirodelae Herba | 64.5 |
| **B5** | 17 | Rosae Laevigatae Fructus | 89.3 | **F5** | 65 | Schizonepetae Herba | 90.2 |
| **B6** | 18 | Tatarian Aster Root | 103.5 | **F6** | 66 | Perillae Caulis | 75.0 |
| **B7** | 19 | Ephedrae Herba | 67.0 | **F7** | 67 | Chaenomelis Fructus | 76.5 |
| **B8** | 20 | Polygalae Radix | 91.3 | **F8** | 68 | Stellariae Radix | 89.8 |
| **B9** | 21 | Stemonae Radix | 89.8 | **F9** | 69 | Phragmitis Rhizoma | 85.0 |
| **B10** | 22 | Semen Arecae (*Areca catechu* L.) | 50.0 | **F10** | 70 | Poriae Cutis | 61.6 |
| **B11** | 23 | Cistanches Herba | 101.5 | **F11** | 71 | Isatidis Radix (*Isatis indigotica* Fort.) | 52.9 |
| **B12** | 24 | Nelumbinis Plumula | 58.2 | **F12** | 72 | Control | 100 |
| **C1** | 25 | Scutellariae Radix | 70.3 | **G1** | 73 | Peucedani Radix | 78.0 |
| **C2** | 26 | Ophiopogonis Radix | 65.8 | **G2** | 74 | Angelicae Sinensis Radix | 67.0 |
| **C3** | 27 | Cynanchi Atrati Radix Et Rhizoma | 72.6 | **G3** | 75 | Persicae Semen | 99.3 |
| **C4** | 28 | Aconiti Kusnezoffii Radix Cocta | 92.1 | **G4** | 76 | Astragali Radix Praeparata Cum Melle | 87.1 |
| **C5** | 29 | Dioscoreae Nipponicae Rhizoma | 80.9 | **G5** | 77 | Gastrodiae Rhizoma | 74.0 |
| **C6** | 30 | Sargentodoxae Caulis | 56.5 | **G6** | 78 | Cremastrae Pseudobulbus | 67.2 |
| **C7** | 31 | Lonicerae Japonicae Flos | 82.9 | **G7** | 79 | Puerariae Thomsonii Radix | 84.1 |
| **C8** | 32 | Rosin | 73.6 | **G8** | 80 | Sanguisorbae Radix | 74.5 |
| **C9** | 33 | Corni Fructus | 89.1 | **G9** | 81 | Allii Macrostemonis Bulbus | 86.6 |
| **C10** | 34 | Saposhnikoviae Radix | 57.6 | **G10** | 82 | Cassiae Semen | 81.2 |
| **C11** | 35 | Lablab Semen Album | 66.8 | **G11** | 83 | Citri Reticulatae Pericarpium | 85.4 |
| **C12** | 36 | Ampelopsis Radix | 83.7 | **G12** | 84 | Control | 100 |
| **D1** | 37 | Anemarrhenae Rhizoma | 82.6 | **H1** | 85 | Paeoniae Radix Rubra | 75.9 |
| **D2** | 38 | Tribuli Fructus (*Tribulus terrestris* L.) | 46.8 | **H2** | 86 | Angelicae Pubescentis Radix | 63.2 |
| **D3** | 39 | Cirsii Herba | 53.8 | **H3** | 87 | Herb of Hygrometric Boea | 69.1 |
| **D4** | 40 | Acanthopanacis Cortex | 61.6 | **H4** | 88 | Gentianae Macrophyllae Radix | 80.7 |
| **D5** | 41 | Platycodonis Radix | 83.8 | **H5** | 89 | Achyranthis Bidentatae Radix | 87.0 |
| **D6** | 42 | Selaginelle Herba  (*Selaginella tamariscina* (P. Beauv.) Spring) | 5.0 | **H6** | 90 | Dendrobii Caulis | 84.3 |
| **D7** | 43 | Gentianae Radix Et Rhizoma | 83.0 | **H7** | 91 | Asparagi Radix | 87.7 |
| **D8** | 44 | Pulsatillae Radix | 80.7 | **H8** | 92 | Lonice Raejaponicae Caulis | 80.4 |
| **D9** | 45 | Amomi Fructus Rotundus | 87.9 | **H9** | 93 | Plantaginis Semen | 79.9 |
| **D10** | 46 | Astragali Radix | 90.2 | **H10** | 94 | Arecae Pericarpium | 76.6 |
| **D11** | 47 | Citri Reticulatae Semen | 70.1 | **H11** | 95 | Lycii Fructus | 89.6 |
| **D12** | 48 | Rubi Fructus | 98.2 | **H12** | 96 | Control | 100 |
